# Arterial stiffness and COVID‐19: A bidirectional cause‐effect relationship

**DOI:** 10.1111/jch.14259

**Published:** 2021-05-05

**Authors:** Sahrai Saeed, Giuseppe Mancia

**Affiliations:** ^1^ Department of Heart Disease Haukeland University Hospital Bergen Norway; ^2^ University of Milano‐Bicocca, Milano and Policlinico di Monza Monza Italy

## 1 INTRODUCTION

Coronavirus disease 2019 (COVID‐19) primarily affects the lungs but the clinical manifestations vary markedly among the patients involved. In more severe cases, COVID‐19 can lead to acute respiratory distress syndrome (ARDS), multi‐organ failure including cardiac injury and toxic shock syndrome with need for intensive care and a ventilator support,^1^ while at the opposite extreme the signs and symptoms can be those of a mild flu and in many patients the infection is asymptomatic.

Since the outbreak of the infection in China, a large number of studies have consistently reported on the cardiovascular (CV) complications of COVID‐19 which include acute cardiac injury, cardiac arrhythmias, and diffuse endothelial damage leading to microvascular thrombosis and thromboembolic events. Evidence has also consistently suggested that cardiac, vascular, renal, and cerebral damages associated with CV risk factors such as hypertension, diabetes, dyslipidemias, and obesity markedly increase the risk of COVID‐19 progression to its most severe and lethal forms, presumably because the pre‐existing structural and functional alterations of the organs affected by long‐lasting blood pressure elevation and metabolic alterations weaken their resistance to the virus. Resistance to the virus may be further reduced by the pathogenetic nature of some of these organ alterations, that is, by the fact the most common cause of risk factor‐dependent complications, atherosclerosis, is likely to be co‐determined by an inflammatory process that may adversely interact with that of the coronavirus.^1^ As far as obesity, a major component of the metabolic syndrome, is concerned, its relationship with the COVID‐19 severity may be due to its association with chronic low grade inflammation, higher leptin and lower adiponectin levels, immune response dysregulation, and abnormal pro‐inflammatory cytokine production.^2^

The association between increased arterial stiffness and the severity and duration of chronic inflammation in a number of systemic inflammatory diseases are well established.^3^ The vascular structure and function are altered by edema and inflammatory cells. Prior studies have supported the cross talk between inflammation and arterial stiffness by showing that patients with chronic inflammatory diseases such as rheumatoid arthritis and chronic inflammatory bowel disease have higher arterial stiffness than control subjects matched for age, sex and blood pressure.^4^ Hence, the severity of underlying chronic inflammation in these disease states goes hand‐in‐hand with the severity of arterial stiffness. For all these interactive factors, there is the basis for a vicious circle because, by increasing the traumatic effect of intravascular pulsatile pressure on the vascular wall, arterial stiffening favors atherosclerosis with an increase in its inflammatory component and a further stiffening effect. The same may be true for COVID‐19. COVID‐19 is a multisystem disease with hyperinflammation and altered immune response and may therefore have detrimental effects on the systemic vasculature both on short and long run. A practical example may be the ethnic minorities who experience the more aggressive forms of COVID‐19 with an enhanced systemic inflammatory response and higher rates of CV complications.^5^ Of note, these patients have a substantial higher prevalence of pre‐existing hypertension, obesity, and diabetes, a cluster which is associated with systemic inflammation and may weaken the resistance of target organs against the coronavirus.

During the COVID‐19 pandemic, CV research has reserved little attention to vascular damage such as increased large artery stiffness. This has little justification because large artery stiffening has been repeatedly shown to have a paramount clinical importance because it: 1) predicts future progression to hypertension in people who exhibit a normal BP^6^, ^7^; 2) increases the long‐term risk of future events and death, independently on the contribution of other risk factors^8^; and 3) favors damage of organ structure and function by increasing pulsatile flow transmission to the microvasculature. The possibility that large artery stiffening is part of the CV phenotype of COVID‐19 is also supported by the evidence that some factors which are known to operate during the COVID‐19 disease are known to adversely affect large artery distensibility, which will be discussed below.

## 2 PREVIOUS WORK

During the COVID‐19 pandemic, there have been so far only few small studies assessing the impact of COVID‐19 on arterial stiffness (Table 1). As an initial contribution to the field, Schnaubelt *et al* recently showed in a small study of 22 COVID‐19 patients compared with 22 age‐ and sex‐matched controls, that COVID‐19 was independently associated with increased arterial stiffness as reflected by increased carotid‐femoral or brachial‐ankle pulse wave velocity (PWV).^9^ Median carotid‐femoral PWV was 14.3 m/s in acutely ill COVID‐19 patients and 11.0 m/s (*P* =.007) in age‐ and sex‐matched non‐COVID‐19 patients. Similarly, median brachial‐ankle PWV was 19.9 m/s in COVID‐19 patients and 16.0 m/s (*P* =.019) in controls. Both carotid‐femoral (*P* =.056) and brachial ankle (*P* =.004) PWV were also higher in non‐survivors of COVID‐19 than in COVID‐19 survivors. PWV correlated with the length of hospital stay. Furthermore, some other preliminary studies have also reported on the vascular implications of COVID‐19 infection.^10^, ^11^ In a small, cross‐sectional study, Ratchford *et al* showed that young adults who 3‐4 weeks prior to vascular health assessment tested positive on COVID‐19, had significantly impaired vascular function as reflected by lower flow‐mediated dilation (FMD) of the brachial artery, and increased arterial stiffness derived from carotid‐femoral PWV, compared with young healthy adults without prior COVID‐19 infection.^10^ FMD expressed as percentage change was 2.71 ± 1.21% in the COVID‐19 group and 8.81 ± 2.96% (*P* <.01) in the control group. Carotid‐femoral PWV was 5.83 ± 0.62 m/s in COVID‐19 group and 5.17 ± 0.66 m/s (*P* <.01) in the control group.

**TABLE 1:** Overview of the studies assessing arterial stiffness and function in COVID‐19

First author (reference no)	Mean age	Number of patients and controls	Design	Method for assessment of vascular health	Major findings
Schnaubelt *et al* [9]	76.5 years	22 COVID‐19 patients 22 non‐COVID‐19 controls	Case control	Brachial‐ankle and carotid‐femoral PWV	COVID‐19 patients had higher PWV than controls (14.3 m/s vs. 11.0 m/s, *P* =.007). In COVID‐19 survivors (n = 11), PWV correlated with the length of hospital stay (mean 12.6 ± 4.3 days). PWV was higher in non‐survivors of COVID‐19 than in COVID‐19 survivors.
Ratchford *et al* [10]	COVID‐19: 20 years Control: 23 years	11 COVID‐19 patients 20 controls	Cross‐sectional Case control	Brachial FMD Leg sPLM Carotid‐femoral PWV	COVID‐19 patients had impaired vascular function and higher arterial stiffness compared with control group: ‐ lower FMD of the brachial artery (2.71 ± 1.21% vs. 8.81 ± 2.96%, *P* <.01). ‐ higher carotid‐femoral PWV (5.83 ± 0.62 m/s vs. 5.17 ± 0.66 m/s, *P* <.01)
Rodilla *et al* [11]	67.5 years	12 170 patients (2606 non‐survivors, 9564 survivors)	Retrospective All‐cause mortality at 50 days	Admission pulse pressure ≥60 mmHg	Increased arterial stiffness (admission pulse pressure ≥60 mmHg) was associated with higher risk for all‐cause mortality in COVID‐19 patients (adjusted odds ratio 1.27, *P* =.0001)

Abbreviations: FMD, flow‐mediated dilation; PWV, pulse wave velocity; sPLM, single passive limb movement.

Finally, Rodilla *et al* showed that a pulse pressure of ≥60 mmHg at admission, as a surrogate marker of arterial stiffness, was associated with higher risk of all‐cause mortality (adjusted odds ratio 1.27, *P* =.0001) in hospitalized COVID‐19 patients.^11^ In addition, in an individual case study involving a 55‐year‐old man with obesity and no previously known hypertension, we recently showed that several months after recovery from COVID‐19, the patient had sustained tachycardia and elevated blood pressure at rest. We speculated that the activation of renin‐angiotensin and sympathetic systems, inflammation‐induced systemic cytokine, volume overload and associated hyperreninemia, inflammation, and vasculitis may have contributed to an exaggerated cardiovascular response and sustained tachycardia at rest.^12^ However, whether this persistently elevated heart rate and blood pressure was a consequence of COVID‐19‐induced increased arterial stiffness, or an untreated persistent abnormal CV response is the primary responsible factor for an incident increased arterial stiffness, is a matter for well‐designed prospective studies on COVID‐19 survivors to clarify.

## 3 POSSIBLE MECHANISMS OF ENDOTHELIAL DAMAGE IN COVID‐19

**FIGURE 1:**
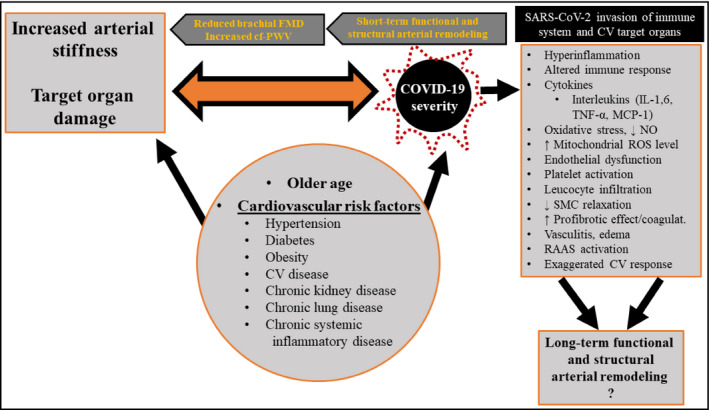
The bidirectional association between baseline increased arterial stiffness and target organ damage, and COVID‐19 severity. Both target organ damage and COVID‐19 disease severity share the same risk factors. Through a number of possible mechanisms, COVID‐19 can cause short term functional and structural remodeling of the vascular wall. However, the long‐term effects are yet to be seen. CV, cardiovascular; cf‐PWV, carotid‐femoral pulse wave velocity; FMD, flow‐mediated dilation; IL, interleukin; MCP‐1, monocyte chemoattractant protein 1; NO, nitric oxide; RAAS, renin‐angiotensin‐aldosterone system; SMC, smooth muscle cell; TNF‐α, tumor necrosis factor alpha

Endothelial dysfunction is believed to be a key element in the pathogenesis of COVID‐19‐related organ damage.^13^, ^14^, ^15^, ^16^ Both endothelial dysfunction and COVID‐19 severity and associated inflammation have a direct bilateral correlation and accelerates each other.^14^, ^17^ The COVID‐19‐dependent direct endothelial damage is believed to cause subintimal inflammation, hemorrhage and thrombosis, dysregulating vascular tone, causing edema, increasing the matrix metalloproteinase levels and leading to functional and structural arterial remodeling.^18^ Indeed, large artery stiffening may in principle be one of the COVID‐19 damages with a greater chance to persist after recovery from COVID‐19, a sequela that may materialize if the post‐infection fibrosis that has been documented in the lungs extends to the vascular wall tissue, that is, if in the large artery wall fibrosis replaces the elastic tissue component. The potential mechanisms that may adversely affect arterial function and structure in COVID‐19 disease are shown in Figure 1.

Another possible mechanism is the modification of the arterial wall tissue brought about by the cytokine‐dependent increase of inflammatory cells and of the inflammatory edema, given the repeated demonstration of a close relationship between arterial stiffness and the severity and duration of chronic inflammation in a number of systemic inflammatory diseases.^3^, ^4^ Systemic cytokine storm as result of an aggressive systemic inflammation in COVID‐19 and dysregulation of immune response have gained much attention during the pandemic.^1^ This may also lead to endothelial dysfunction, increased oxidative stress, decreased nitric oxide secretion and bioavailability, decreased relaxation of smooth muscle cells and vasoconstriction with an increase in the elastic modulus compared to the relaxed state. Increased blood viscosity due to hyperglycemia, hypoxia, disseminated intravascular coagulation (DIC), and enhanced fibrinogen degradation products are other contributing factors (Figure 1).^19^ Upregulation of inflammatory cytokines such as tumor necrosis factor alpha (TNF‐α) and interleukins (ILs) (IL‐6 and IL‐8), enhance platelet reactivity and activation of leukocytes to form neutrophil extracellular traps (NETs) which further contributes to a pro‐thrombotic state​ and formation of microvascular thrombi.^20^, ^21^ IL‐6 also causes detrimental endothelial activation which increases vascular permeability through nitric oxide.^15^

Other theories such as SARS‐CoV‐2 infection‐related mitochondrial dysfunction and increase inmitochondrial reactive oxygen species (ROS) production have also been proposed.^22^ Mitochondrial ROS functions as signaling molecule keeping basal vascular hemostasis and inflammatory pathways in balance. Enhanced ROS production by dysfunctional mitochondria leads to oxidative stress and promotion of inflammation and chronic endothelial dysfunction with subsequent CV disease and lung injury after recovery from COVID‐19 have also been proposed.^22^ It has been also postulated that mitochondrial ROS may induce premature aging through oxidative telomere damage.^22^

## 4 THE ROLE OF PRE‐EXISTING ATHEROSCLEROSIS/INCREASED ARTERIAL STIFFNESS IN COVID‐19 SEVERITY

In atherosclerosis, that is, the most important intermediate step between CV risk factors and CV morbid and fatal events, endothelial cells are damaged and the blood flow patterns are irregular. These lead to subendothelial plasma lipoprotein (LDL) deposition and oxidation, plaque formation, and narrowing of the artery lumen. Pre‐existing atherosclerosis is an independent risk factor for COVID‐19 severity, which is evident from the studies reporting higher mortality in COVID‐19 patients with underlying CV risk factors and morbidities. Vinciguerra *et al* hypothesized that in COVID‐19 patients with pre‐existing atherosclerosis, an immune system dysregulation favors SARS‐CoV‐2 entry into human cells. However, atherosclerosis may also be a long‐term consequence of COVID‐19 by activation and initiation of chronic vascular endothelial dysfunction.^23^ Data from the UK Biobank have recently demonstrated that COVID‐19‐positive individuals who died had significantly lower left ventricular stroke volume, more often impaired global longitudinal strain (GLS), and lower arterial compliance (lower aortic distensibility and higher arterial stiffness index) compared to those who survived.^24^

## 5 POST‐COVID‐19 EARLY ATHEROSCLEROSIS/INCREASED ARTERIAL STIFFNESS

Whether COVID‐19 patients are predisposed to early atherosclerosis is not yet fully understood. However, it is reasonable to believe that post‐COVID‐sequelae such as worsening of hypertension, diabetes, renal damage, and inflammation‐induced dyslipidemia (altered lipid metabolism) and endothelial dysfunction may contribute to early atherosclerosis and accelerate vascular aging. Furthermore, sympathetic nerve activity, dysregulation of the RAS, altered ACE‐2 expression may lead to adverse structural and functional remodeling of the arteries, and therefore, a bidirectional association between pre‐existing arterial stiffness and COVID‐19 severity, as well as post‐COVID increase in arterial stiffness, can be seen (Figure 1). Even lack of physical activity and psychological stress during lockdown may be important functional modulators of arterial stiffness. Hence, increased arterial stiffness at baseline due to age or CV risk factors and comorbidities is associated with more severe COVID‐19 disease, and COVID‐19 per se may be associated with further acceleration of arterial stiffening (arterial sclerosis) or development of early atherosclerosis. Although some preliminary reports, as mentioned earlier, support these speculations, this need to be proven in future studies, and is of great clinical importance for public health in terms of early detection and prevention of atherosclerosis and reducing the risk of early vascular events such as coronary artery disease and stroke in these vulnerable patients.

Statin lower CV risk factors through their lipid‐lowering and pleiotropic or anti‐inflammatory effects.^25^ It has been also postulated that statin may act as a SARS‐CoV‐2 main protease inhibitor.^26^ Some preliminary reports have shown that use of statin in COVID‐19 patients is associated with improved survival rate^27^ although this also requires further validation in other studies. Furthermore, the role of anti‐inflammatory drugs such as colchicine, nitric oxide, and heparin in COVID‐19 patients will be explored by some ongoing studies.^28^

## 6 CONCLUSION

There appears to be a bidirectional association between arterial stiffness/atherosclerosis and COVID‐19 severity. Preliminary studies have already documented the association of COVID‐19 with adverse functional and morphological changes in the arterial wall. Endothelial damage by direct invasion of the coronavirus or through inflammation‐induced systemic cytokine storm is believed to be a key element in the pathogenesis of COVID‐19‐related target arterial damage.

## 7 PERSPECTIVES

We should acknowledge that this opinion piece is prepared from a clinical point of view and hence hypothesis generating. The molecular mechanism is not discussed, although these are still poorly understood. Therefore, larger prospective studies with assessment of arterial stiffness in patients with or without prior COVID‐19 infection should be performed to gain more information on the acute and chronic impact of COVID‐19 infection on systemic vasculature, particularly in terms of developing early atherosclerosis or an unsuccessful early vascular aging. The establishment of larger collaborative projects is essential to examine the long‐term vascular consequences of COVID‐19 and reduce the risk of vascular events in COVID‐19 survivors. Hopefully, future longitudinal studies will also examine the prognostic impact of baseline left ventricular hypertrophy and increased PWV in COVID‐19 patients. There are very limited blood pressure data, particularly involving ambulatory blood pressure monitoring after recovery from COVID‐19. This should be the focus of future vascular research in COVID‐19.

​

## CONFLICT OF INTEREST

No conflict of interest.

## AUTHOR CONTRIBUTIONS

Sahrai Saeed and Giuseppe Mancia contributed to the conception of this work. Sahrai Saeed wrote the first draft. Giuseppe Mancia critically revised it for important intellectual. Both authors approved the final submission.
